# Novel Agents for the Treatment of Multiple Myeloma: Proteasome Inhibitors and Immunomodulatory Agents

**Published:** 2013-09-01

**Authors:** Sandra E. Kurtin, Elizabeth Bilotti

**Affiliations:** From University of Arizona Cancer Center, Tucson, Arizona, and John Theurer Cancer Center at Hackensack University Medical Center, Hackensack, New Jersey

## Abstract

The integration of novel agents into the treatment of multiple myeloma (MM) has shifted the focus from an incurable disease to one that is chronic, with a realistic hope of someday achieving a cure. Proteasome inhibitors and immunomodulatory agents are the backbone of novel therapies for MM. These agents are particularly important for patients with relapsed or refractory disease, a fate faced by the majority of myeloma patients over the course of their disease. Review of recent clinical trial data for the proteasome inhibitors and immunomodulatory agents, including clinical efficacy and safety information, will assist the advanced practitioner in oncology with integrating these data into the current treatment guidelines for MM.

Multiple myeloma (MM) accounts for only 1% of all malignancies but is the second most common hematologic malignancy, with approximately 21,700 cases diagnosed each year and approximately 10,710 deaths expected in the United States in 2013 (American Cancer Society, 2013). The average age at diagnosis is 69 years. Although MM is not curable, the median overall survival has improved dramatically over the past decade as a result of clinical trials utilizing novel agents in the treatment of all stages of MM. These trials have improved the understanding of the pathobiology of MM and have helped to identify attributes of the malignant clone and the tumor microenvironment, which may provide new therapeutic targets (Palumbo & Anderson, 2011). Achievement of an early and deep response followed by a sustained response with an acceptable level of toxicity is considered to be the best outcome for treatment of MM and is associated with improved long-term survival (Palumbo & Cavallo, 2012).

Proteasome inhibitors and immunomodulatory agents, which are among the novel agents thought to explain the improvement in clinical outcomes for patients with MM, will be the focus of this article. It is important to note that the clinical trial endpoints for the studies discussed vary based on individual trial design and should interpreted within that context. The National Comprehensive Cancer Network (NCCN, 2013) provides guidelines based on analysis of current scientific data by a panel of experts within its membership organizations. Additional treatment guidelines have been suggested by consensus groups such as the International Myeloma Foundation and the Multiple Myeloma Research Foundation. Familiarity with risk-adapted treatment selection, desired clinical outcomes, and the current role of novel agents in the treatment of MM is necessary to effectively incorporate these agents into the treatment paradigm for MM (Tables 1 through 4). Familiarity with the safety and clinical efficacy profile, clinical management guidelines, and patient and caregiver education specific to proteasome inhibitors and immunomodulatory agents will provide the necessary tools for effectively integrating these agents into the treatment plan for patients living with MM.

**Table 1:**
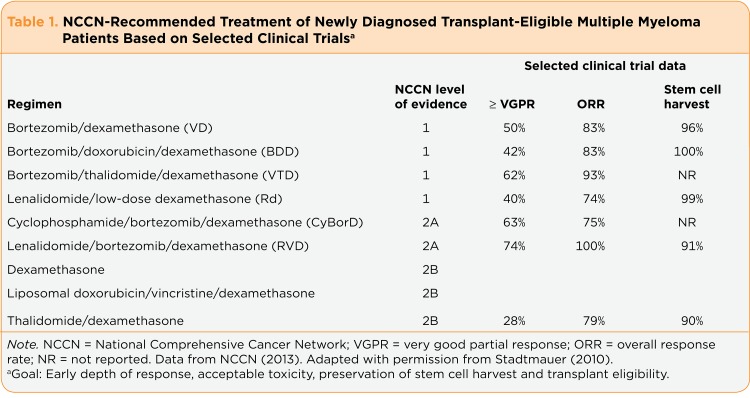
Table 1. NCCN-Recommended Treatment of Newly Diagnosed Transplant-Eligible Multiple Myeloma Patients Based on Selected Clinical Trials

**Table 2:**
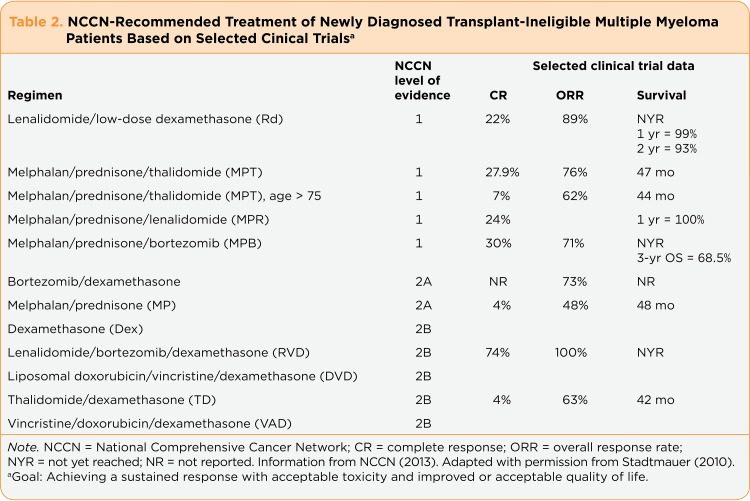
Table 2. NCCN-Recommended Treatment of Newly Diagnosed Transplant-Ineligible Multiple Myeloma Patients Based on Selected Cinical Trials

**Table 3:**
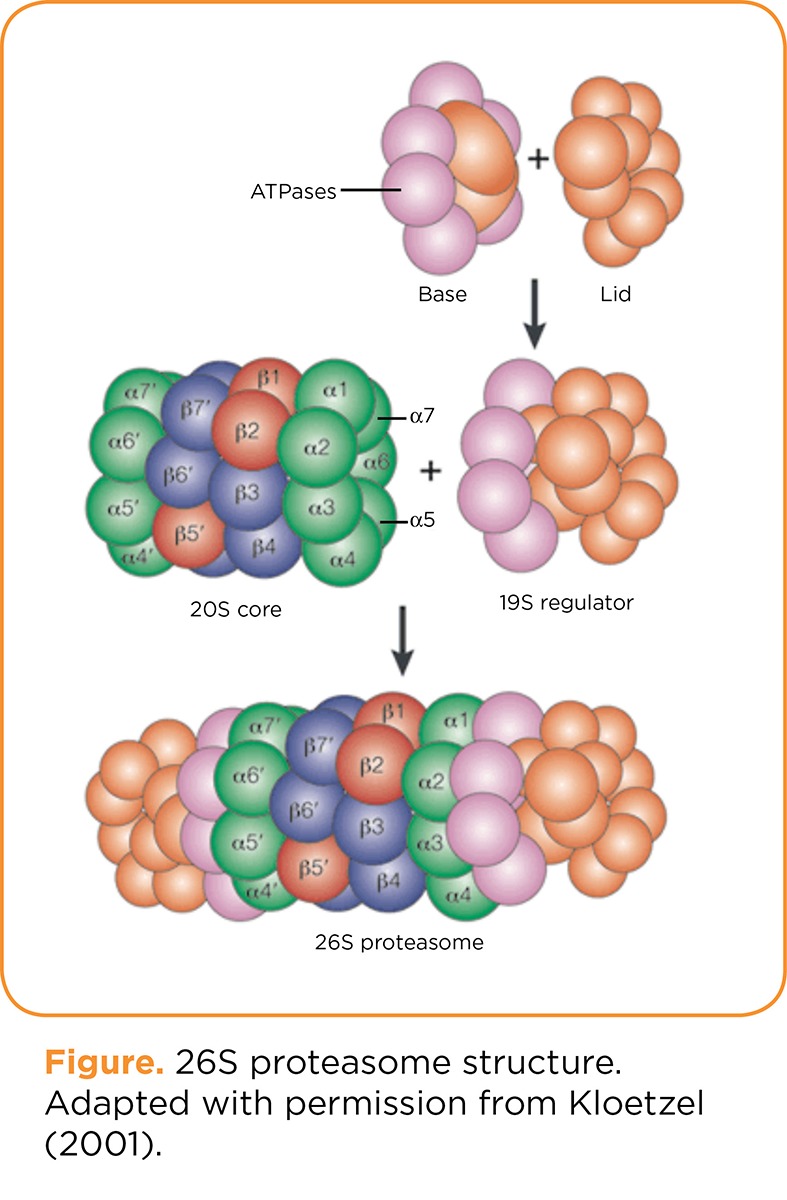
Table 3. NCCN-Recommended Maintenance Therapy Following Stem Cell Transplant or Continuous Treatment in Transplant-Ineligible Patients With Multiple Myeloma

**Table 4:**
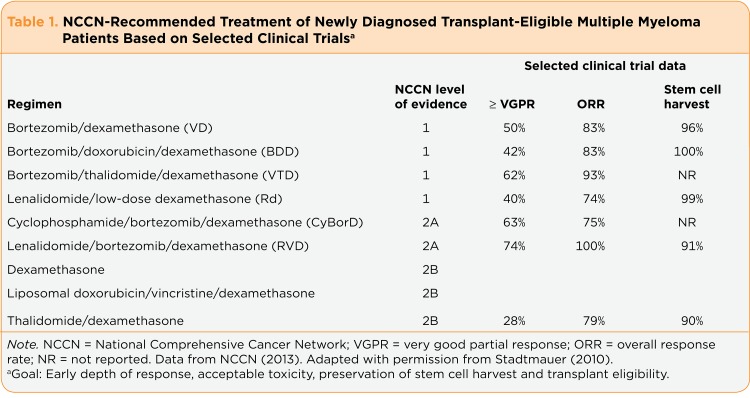
Table 4. Current FDA-Approved Options for Salvage Treatment in Patients With Relapsed or Refractory Multiple Myeloma Based on Selected Clinical Trials

## Proteasome Inhibitors

The proteasome is an intracellular protein complex responsible for the breakdown of regulatory proteins within the cell, including those that regulate cell-cycle progression, apoptosis, and DNA repair (Adams, 2004); see Figure. The proteolytic cleavage of ubiquitinated proteins within the proteasome core can occur at one or more of three identified subunits: â1 (caspase-like activity), â2 (trypsin-like activity), and â5 (chymotrypsin-like activity); see Table 5. Ultimately, the inhibition of proteasome activity leads to growth arrest and apoptosis, which is particularly important in cancer cells, as they often have a higher level of proteasome activity with an increase in sensitivity to the inhibitory effects when compared with normal cells (Adams, 2004).

**Figure 1:**
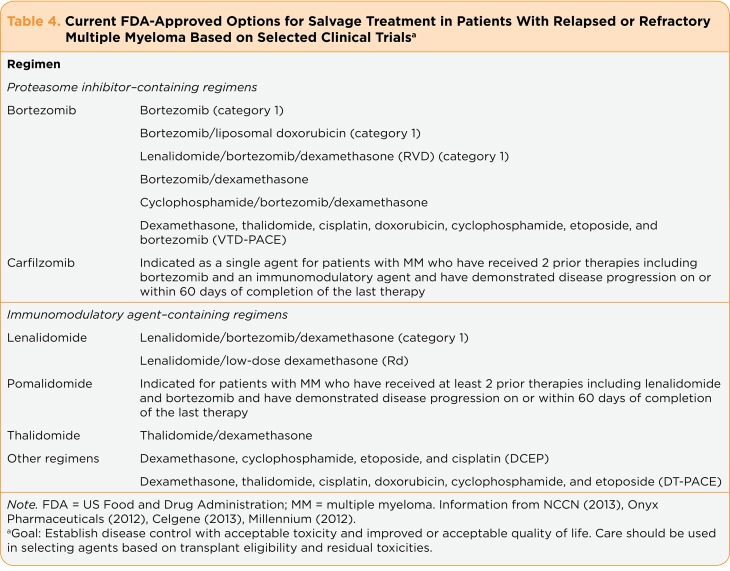
Figure. 26S proteasome structure. Adapted with permission from Kloetzel (2001).

**Table 5:**
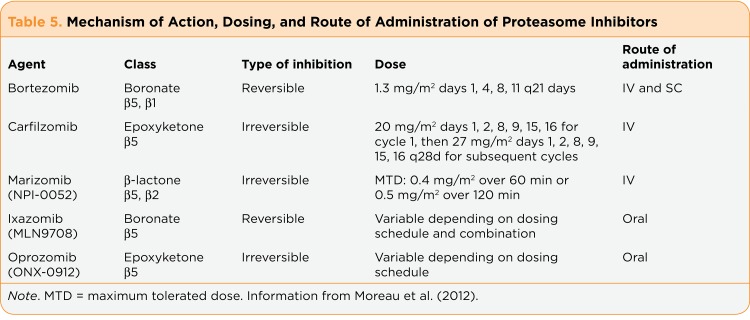
Table 5. Mechanism of Action, Dosing, and Route of Administration of Proteasome Inhibitors

## BORTEZOMIB

Bortezomib (Velcade) is a first-in-class reversible proteasome inhibitor that originally received accelerated review by the US Food and Drug Administration (FDA) based on the results of the phase II SUMMIT trial establishing safety and efficacy of single-agent bortezomib vs. pulse dexamethasone in patients with relapsed MM who had received one to three prior lines of therapy (Richardson et al., 2003). The initial findings showed a significant improvement in time to disease progression, with improvements in overall response rate (ORR) and survival (Richardson et al., 2003). Subsequent and final analyses at 22 months of follow-up showed a 6-month improvement in overall survival (OS; 30 vs. 24 months), significant improvement in ORR (43% vs. 18%), improved depth of response (complete response [CR] rate of 9% vs. < 1%), and a 2.7-month improvement in time to disease progression (TTP; Richardson et al., 2007).

San Miguel and colleagues reported data from the randomized phase III VISTA trial evaluating bortezomib in combination with oral melphalan and prednisone (VMP) vs. oral melphalan and prednisone (MP) in non–transplant-eligible newly diagnosed MM patients (San Miguel et al., 2012). At a median follow-up of 60.1 months, there was a 31% reduced risk of death with VMP vs. MP (hazard ratio [HR], 0.695;* p* = .001; median OS, 56.4 vs. 43.1 months). Time to next therapy (median, 30.7 vs. 20.5 months; HR, 0.557;* p* = .001) was longer with VMP than with MP. The analysis also found that this benefit extended to elderly patients (³ 75 years), those with International Staging System (ISS) stage III disease, and those with renal impairment (creatinine clearance [CrCl] < 60 mL/min; San Miguel et al., 2012).

Moreau and colleagues (2011) reported results of a noninferiority study comparing the efficacy of bortezomib as an intravenous (IV) push with a subcutaneous (SC) injection administered with the same dose and schedule in bortezomib-naive relapsed MM patients who had received one to three prior therapies (Moreau et al., 2011). There were no differences in ORR, depth of response, or time to response between the two study arms; however, the incidence of peripheral neuropathy (PN) was significantly reduced in the cohort treated with SC bortezomib (38% vs. 53% all grades, 6% vs. 16% grade 3 and higher; Moreau et al., 2011). The results of this trial have led to a change in the favored route of administration from IV to SC and a change in the standard schedule of administration to a twice-weekly dosing schedule for two cycles, followed by weekly dosing. Together, these changes offer similar efficacy, the opportunity to improve treatment outcomes with continued therapy, and improved quality of life by lessening toxicity (Moreau, 2012).

## CARFILZOMIB

Carfilzomib (Kyprolis) is a second-in-class irreversible proteasome inhibitor approved for the treatment of patients with MM who have received at least two prior therapies including bortezomib and an immunomodulatory agent and have demonstrated relapsed or refractory disease (Onyx Pharmaceuticals, 2012). Relapsed or refractory disease is defined as disease progression on or within 60 days of completion of the last therapy.

The phase II PX-171-003-A1 study established safety and efficacy of single-agent carfilzomib in the treatment of patients with relapsed and/or refractory MM (n = 257, median age 63 years; Siegel et al., 2012). The median number of prior lines of therapy was 5. A total of 74% of patients had documented disease progression on their most recent line of therapy. All but one patient had received bortezomib, and all patients had received an immunomodulatory agent. A total of 73% of the patients were refractory to bortezomib; 80% were refractory or intolerant (therapy discontinued due to toxicity) to both bortezomib and lenalidomide.

The ORR in the heavily pretreated population was 23.7% (61/257) for the response-evaluable patients, with a median duration of response of 7.8 months (Siegel et al., 2012); see Table 6. Patients meeting the eligibility criteria for this trial are similar to those analyzed by Kumar et al. (2012b), where patients who were identified as refractory to bortezomib and either refractory to, intolerant to, or ineligible for immunomodulatory therapy had an expected OS of 9 months once identified (Kumar et al., 2012b). The efficacy results of the single-agent carfilzomib trial are promising in this patient population.

**Table 6:**
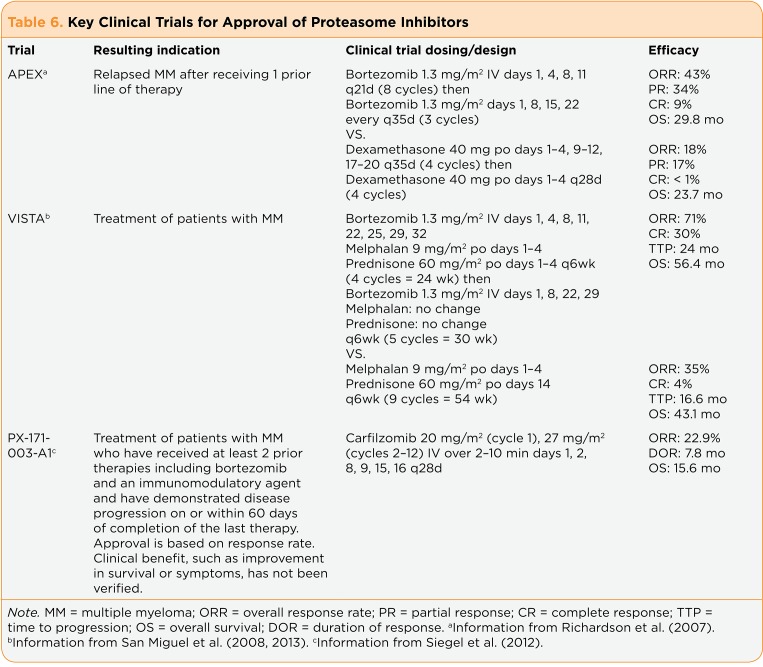
Table 6. Key Clinical Trials for Approval of Proteasome Inhibitors

Pooled data from 526 patients with MM enrolled in four phase II single-agent carfilzomib trials (PX-171-003-A0, PX-171-003-A1, PX-171-004, and PX-171-005) were analyzed for various safety endpoints. The first analysis focused on the hematologic toxicities, both overall and grade 3/4. It was found that the thrombocytopenia was cyclical, similar to bortezomib, with the nadir on day 8 of the treatment cycle (Nooka et al., 2012). No clinically significant bleeding episodes (< 1% grade 3 and no grade 4) were associated with concurrent thrombocytopenia. Only a 1% incidence of febrile neutropenia was reported, despite a reported grade 3/4 neutropenia incidence of 12%. Anemia was reported at 46.8% for all grades, with 22.4% grade 3/4 (Nooka et al., 2012).

The second analysis focused on renal complications, as this is a common concern when treating patients with MM. Moderate to severe renal dysfunction (CrCl < 50 mL/min) was reported in 23.8% of patients at the time of study entry (Harvey et al., 2012). Worsening renal function was transient in 6% of patients (average duration 1.4 weeks) and sustained in 7% of patients; however, only 1.5% of patients required treatment discontinuation due to renal dysfunction (Harvey et al., 2012). As the PX-171-005 data reported no difference in the pharmacokinetics, safety, or efficacy when carfilzomib was used in patients with varying degrees of renal impairment, including those on dialysis, no dose adjustment is indicated (Niesvizky et al., 2011).

The final subset analysis focused on the incidence of either worsening or treatment-emergent PN, which can be a dose-limiting toxicity of proteasome inhibitor therapy. A history of PN attributed to prior treatment for MM was reported by 84.8% of patients, with 71.9% of patients having either grade 1 or 2 PN at the time of study entry (Martin et al., 2012). The reported incidence of PN in all carfilzomib studies was 13.9% overall, with 12.5% grades 1/2, 1.3% grade 3, and no grade 4. Dose adjustment (0.8%, n = 4) or discontinuation of carfilzomib (0.2%, n = 1) due to PN was rare (Martin et al., 2012).

Carfilzomib continues to be studied in multiple settings. The current dosing recommendations suggest a body surface area limit of 2.2 m2. Many study designs are evaluating its use in combination with other antimyeloma therapies in both the newly diagnosed and relapsed settings. It is also being evaluated in dose-escalation studies to determine the maximum tolerated dose (MTD) in both solid tumors and hematologic malignancies, including MM.

## PROTEASOME INHIBITORS UNDER INVESTIGATION

Although there are numerous new proteasome inhibitors in the pipeline for both multiple myeloma and other malignancies, there are safety and efficacy data available in a small number of patients from both phase I and II trials for two of these agents.

Marizomib (NPI-0052) is an irreversible proteasome inhibitor administered intravenously. At present, two phase I dose-escalation trials have evaluated safety outcomes in patients with relapsed and refractory MM. Most of the patients enrolled had prior bortezomib exposure, with 71% documented as bortezomib refractory with a median of 6 prior lines of therapy. An ORR of 14% (all partial responses) was reported, with stable disease or better in 73% (Richardson et al., 2011a). The dose-limiting toxicities in those trials were reversible neurologic symptoms, including transient hallucinations, cognitive changes, and loss of balance. This agent continues to be evaluated on a twice-weekly schedule at a dose of 0.5 mg/m^2^ IV over 120 minutes on days 1, 4, 8, and 11 of a 21-day cycle either alone or with low-dose oral dexamethasone 20 mg the day prior to and day of marizomib dosing (Richardson et al., 2011a).

Ixazomib (MLN9708) is an orally administered reversible proteasome inhibitor currently investigated in phase I studies evaluating both weekly and biweekly dosing schedules in relapsed/refractory MM patients. Lonial et al. (2012) reported data from the twice-weekly dosing schedule with ixazomib on days 1, 4, 8, and 11 of a 21-day cycle. The identified MTD was 2 mg/m^2^ (Lonial et al., 2012). Adverse events (AEs) were common (91% of patients reporting at least one AE), with the most frequently reported treatment-emergent adverse events (TEAEs) being thrombocytopenia, neutropenia, fatigue, nausea, diarrhea, and rash. Peripheral neuropathy was mild (10% overall, no grade 3 or greater reported).

Among the 53 evaluable patients, responses were 1 near CR (nCR), 1 very good partial response (VGPR), 3 partial responses (PRs), and 1 stringent CR (sCR) occurring in a bortezomib-naive patient (Lonial et al., 2012). At the same time, Kumar et al. (2012a) reported results from the once-weekly dosing schedule of ixazomib on days 1, 8, and 15 of a 28-day cycle. In this patient population, three dose-limiting toxicities (DLTs) were seen, including one grade 3 rash and two grade 3 GI adverse events, with a MTD of 2.97 mg/m^2^ orally. Treatment response in the 18 evaluable patients included 1 VGPR, 1 PR, and 8 patients with stable disease lasting up to 9.5 months (Kumar et al., 2012a).

A phase I/II study evaluating twice-weekly oral ixazomib in combination with lenalidomide and dexamethasone (oral ixazomib 4 mg on days 1, 8, and 15 with oral lenalidomide 25 mg on days 1 through 21 and oral dexamethasone 40 mg on days 1, 8, 15, and 22 of a 28-day cycle) in newly diagnosed MM patients reported preliminary results (Richardson et al., 2012). Of 64 evaluable patients, combined responses for phase I/II revealed an ORR of 91%, with 39% VGPR or better (Richardson et al., 2012). The most commonly reported TEAEs (³ grade 3) included vomiting, nausea, thrombocytopenia, syncope, lymphopenia, and fatigue. Peripheral neuropathy occurred in 21% of patients, with > grade 3 reported in one patient treated with a dose of ixazomib above the MTD. These trials offer the promise of additional proteasome inhibitors for the management of patients with multiple myeloma.

## Immunomodulatory Agents

The immunomodulatory agents represent a class of drugs with versatile therapeutic properties, including antiproliferative effects on the malignant clone (antitumor effect), immunomodulatory effects (costimulation of T cells, suppression of regulatory T cells [Tregs], and activation of natural killer [NK] cells), and disruption of plasma cell (PC) microenvironment interactions (antiangiogenesis, anti-inflammatory cytokines, downregulation of adhesion molecules, and antiosteoclastogenic properties; Morgan, Walker, & Davies, 2012, Palumbo & Anderson, 2011; Quach et al., 2010); see Table 7. The immunomodulatory agents are analogs of thalidomide (Thalomid), first used therapeutically for the treatment of relapsed/refractory MM in the late 1990s. More recently, clinical trials using lenalidomide (Revlimid) and pomalidomide (Pomalyst), both analogs of thalidomide, have shown clinical efficacy with different toxicity profiles compared with those of thalidomide.

**Table 7:**
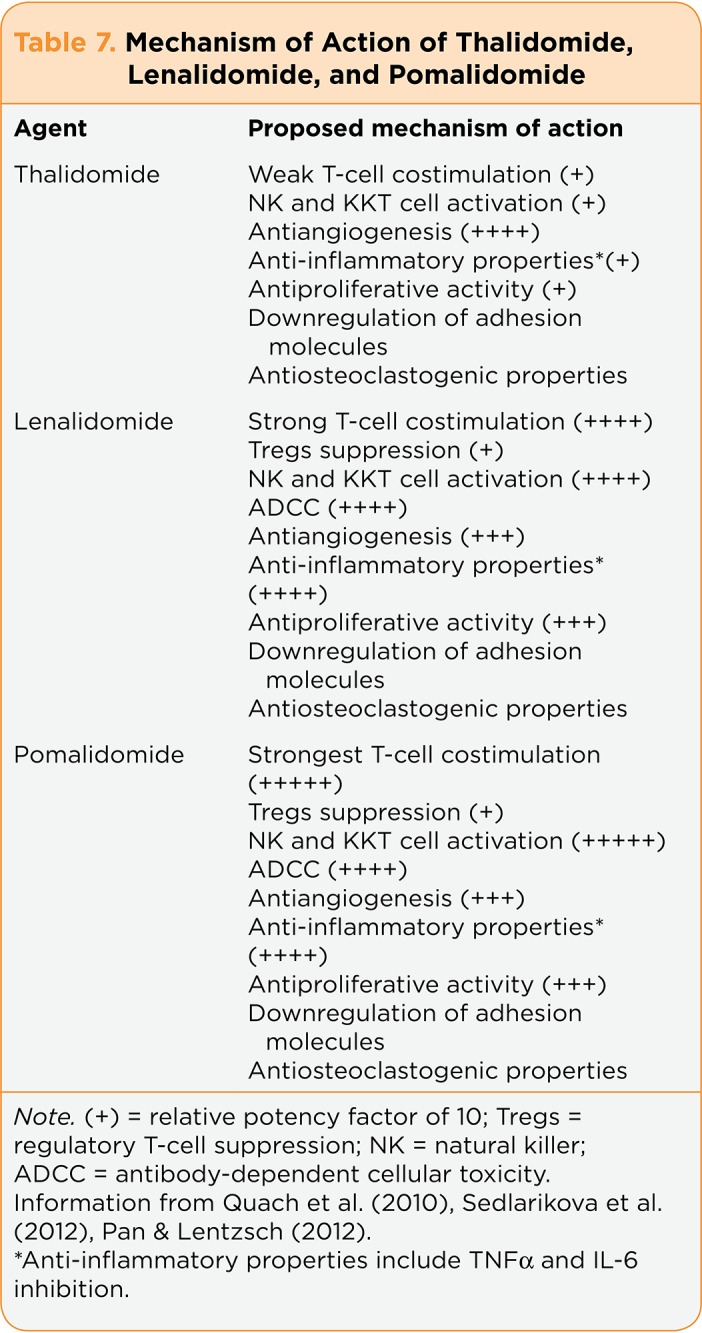
Table 7. Mechanism of Action of Thalidomide, Lenalidomide, and Pomalidomide

Given the pleiotropic mechanism of action, these compounds have been effective in the treatment of MM in combination with dexamethasone as well as in combination with both standard therapies and other novel agents (Quach, Kaiff, & Spencer, 2012; Stewart, 2012; Rajkumar, 2012). As the number of trials conducted over the past decade incorporating thalidomide and lenalidomide in the treatment of MM exceeds 50, with safety and efficacy profiles well established, the focus of this article will be on key trials that have established the conceptual basis for the use of these two agents in specific phases of MM (Tables 1 through 4). Pomalidomide is the newest immunomodulatory agent, approved by the FDA in February 2013. All three agents require dispensing using a safety program due to the historic data documenting the teratogenicity of thalidomide when used as a sedative and antiemetic drug to treat morning sickness in the first trimester of gestation (McBride, 1961; Lenz, 1962). No teratogenic events have been reported in clinical trials to date for either lenalidomide or pomalidomide.

**THALIDOMIDE**

Thalidomide was first reported to have benefit in MM in 1999 in patients with advanced relapsed disease, opening the door for investigating targeted therapies (Singhal et al., 1999). Since that time, numerous trials using thalidomide as a single agent or in combination with dexamethasone or melphalan and prednisone have continued to show benefit. It is currently recommended for the treatment of newly diagnosed patients with MM in both the transplant-eligible and non–transplant eligible populations as well as in patients with relapsed disease (Tables 1 through 4). The chemical structure and clinical experiences of thalidomide were exploited to create the newer immunomodulatory agents lenalidomide and pomalidomide.

**LENALIDOMIDE**

Lenalidomide was approved for the treatment of MM in 2006. E4A03, a large Eastern Cooperative Oncology Group (ECOG) phase III study, established the efficacy and safety of lenalidomide plus high-dose (RD) or low-dose (Rd) dexamethasone in newly diagnosed multiple myeloma. The 1- and 2-year survival rates were 96% and 87%, respectively, for high-dose dexamethasone vs. 88% and 75%, respectively, for low-dose dexamethasone, with subsequent 3-year survival rates of 75% (RD) vs. 74% (Rd). It is important to note that the incidence of deep-vein thrombosis (DVT) was 12% vs. 26% in the Rd and RD arms, respectively. Additionally, the rate of infection in the RD group was higher. These results demonstrated the efficacy and safety of the lenalidomide/dexamethasone combination for previously untreated patients and also raised important questions about the continued use of high-dose dexamethasone vs. the treatment of newly diagnosed multiple myeloma.

Two important phase III trials evaluated the role of lenalidomide/with high-dose dexamethasone vs. high-dose dexamethasone alone in the relapsed setting: MM-009 and MM010. At 48 months of follow-up, pooled analysis of these data confirms superior efficacy of the lenalidomide/dexamethasone arm with improved ORR/CR (61% vs. 15%; * p* < .001), improved TTP (13.4 vs. 4.6 months), and improved OS (38 vs. 31.6 months;* p* = .045). The incidence of thromboembolic events in these trials was 16%, yet prophylactic anticoagulation was not mandatory.

More recent trials have incorporated lenalidomide in combination with other agents such as bortezomib. A phase I/II trial evaluating the combination of lenalidomide, bortezomib, and dexamethasone (RVD) in newly diagnosed MM patients reported response rates of 100%, with 74% VGPR or better (Rajkumar, 2012). Additional trials have evaluated the role of maintenance lenalidomide following autologous stem cell transplantation, showing improvement in event-free survival (EFS); see Tables 1 through 4.

**POMALIDOMIDE**

Pomalidomide, like lenalidomide, is an immunomodulatory compound with pleiotropic properties shown to be beneficial in treating MM. Both agents have been shown to be more potent than thalidomide, with additional immunomodulatory properties thought to enhance the antimyeloma effect, including T-cell costimulation, regulatory T-cell suppression, NK cell activation, and enhanced antibody dependent cell-mediated cytotoxicity (Table 7).

The efficacy of pomalidomide was first established in a small (n = 60) group of relapsed MM patients who had received from 1 to 3 prior therapies, including autologous hematopoietic cell transplantation (Schey et al., 2004). Patients in the first cohort received pomalidomide (POM) 2 mg daily 21/28 days, weekly dexamethasone (LoDEX) 40 mg, and aspirin 325 mg daily. The MTD of pomalidomide in this population was 2 mg, primarily due to myelosuppression, with neutropenia (32% > grade 3) being the most common. Efficacy was established based on a reduction in paraprotein (> 25% in 67%, ³ 50% in 54%, 17% with CR). It is important to note that patients refractory to other novel agents, including lenalidomide (40%), thalidomide (37%), and bortezomib (60%), responded to POM.

A number of trials followed the initial phase I study (Table 8). The majority of these trials combined POM with weekly DEX. Five phase II studies were conducted in sequence by Lacy and colleagues (2009) with variable dosing of POM. The initial cohort of relapsed/refractory MM patients (similar characteristics) received the same regimen as in the phase I trial. Responses included 5% CR, 28% VGPR, and 30% PR. Additionally, responses in patients refractory to other novel agents were reported in 40% of lenalidomide-refractory patients, 37% of thalidomide-refractory patients, and 60% of bortezomib-refractory patients, confirming the role of POM/LoDEX in patients refractory to those agents (Lacy et al., 2009).

**Table 8:**
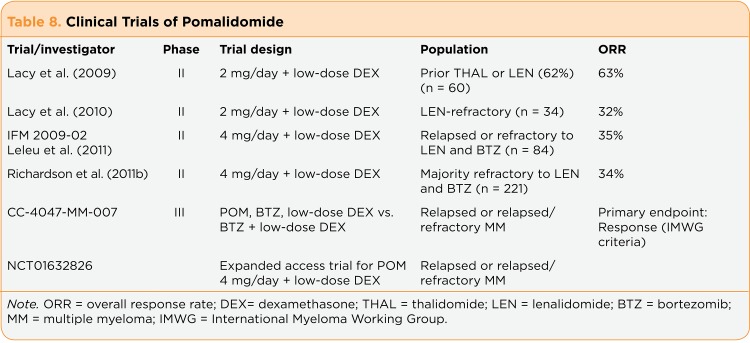
Table 8. Clinical Trials of Pomalidomide

Higher doses of POM in combination with LoDEX in relapsed/refractory MM followed these trials. Lacy and colleagues evaluated three additional cohorts of patients, confirming the efficacy of POM/DEX and concluding that a higher dose of POM (4 mg daily) with DEX did not improve clinical outcomes (Lacy et al., 2011).

Richardson and colleagues (2011b) studied POM (4 mg 21/28)/DEX (40 mg weekly) compared to POM (4 mg 21/28) in a relapsed/refractory MM population (n = 221: POM + LoDEX, n = 113; POM, n = 108). The trial did allow a crossover for patients with progressive disease on the POM-alone arm of the trial. A total of 61 (56%) of these patients went on to receive POM + LoDEX due to progressive disease (PD). Response rates favored the POM/DEX arm of the trial (PR of 34% vs. 13%), with responses seen in patients refractory to novel agents (lenalidomide 30% and bortezomib 16%), suggesting a synergistic effect of the combined regimen. Myelosuppression, in particular grade > 3 neutropenia (38%–47%), was the most common reason for treatment discontinuation.

This trial confirmed the superior efficacy of POM in combination with LoDEX vs. POM alone. In February 2013, the FDA granted accelerated approval to pomalidomide (Celgene, 2013) for the treatment of patients with multiple myeloma who have received at least two prior therapies, including lenalidomide and bortezomib, and have demonstrated disease progression on or within 60 days of completion of the last therapy. As a condition of this accelerated approval, the FDA will require submission of the results of clinical trial CC-4047-MM-007, a randomized trial of pomalidomide added to bortezomib and LoDEX compared to bortezomib plus LoDEX in patients with previously treated multiple myeloma. Pomalidomide is now being evaluated in a number of trials to establish optimal dosing and tolerance in combination with other novel agents (Table 8).

## Toxicities Associated With Proteasome Inhibitors and Immunomodulatory Compounds

Although the proteasome inhibitors and immunomodulatory compounds offer excellent efficacy and additional therapeutic options for patients, familiarity with TEAEs is necessary (Tables 9 and 10). Most patients will receive all agents over the course of their disease with some variability in TEAEs based on both patient and disease-related factors. Each class of agents has some unique TEAEs and these may vary within each class. For example, TEAEs vary within the proteasome inhibitor class due to the differences in targets within the proteasome and the chemical structure of these drugs. Similarly, TEAE profiles vary for the immunomodulatory agents due to the potency of each agent and the secondary changes in the malignant clone and the tumor microenvironment. Each patient must be evaluated prior to initiating therapy for existing comorbid conditions, unresolved toxicities, and risk for new or progressive TEAEs with continued treatment. The goals of therapy and the patient’s wishes must always be considered. Supportive care is essential throughout the continuum of care to minimize the severity of TEAEs. Management strategies for some of the more common side effects of commercially available proteasome inhibitors and immunomodulatory agents will be discussed.

Myelosuppression is a common finding in patients with MM, with anemia often present at the time of diagnosis, while leukopenia/neutropenia and thrombocytopenia emerge more often during treatment and in the relapsed and refractory settings. Familiarity with the incidence, severity, and duration of cytopenias reported in clinical trials for the proteasome inhibitors and immunomodulatory agents will provide a guide for planning the timing and frequency of monitoring blood counts. Guidelines for dose modifications or treatment are provided in the prescribing information for each drug. Supportive care using blood and platelet transfusions or growth factors may be used to prevent more serious AEs at the discretion of the clinician. Pretreatment blood cell counts will determine the feasibility of same-day treatment.

Each patient will need to be evaluated based on their disease status, goals of treatment, bone marrow capacity, comorbid conditions including medications, and general health (Kurtin, 2012). Educating the patient and caregivers on prevention of infections and bleeding, as well as reportable signs and symptoms will allow for prompt intervention and reduce the severity of adverse events (Table 10). Providing recommendations for patients to conserve energy but remain active will minimize the effects of fatigue associated with the disease, treatment, or underlying anemia.

**Table 9:**
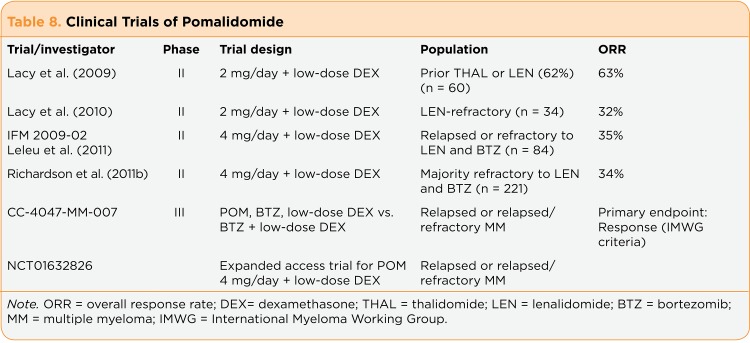
Table 9. Common Adverse Events for Proteasome Inhibitors and Immunomodulatory Agents Used to Treat Multiple Myeloma

**Table 10:**
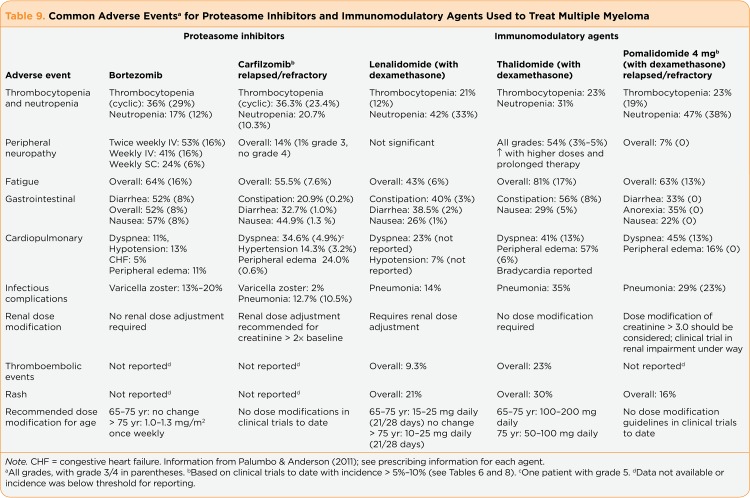
Table 10. Clinical Management of Common Treatment-Emergent Adverse Events Associated With Proteasome Inhibitors and Immunomodulatory Agents Used in the Treatment of Multiple Myeloma

Peripheral neuropathy is a complex process with multiple potential contributing factors in the patient with MM, including the disease itself, diabetes, endocrine disorders, nutritional diseases, vascular disease, connective tissue disease, medications, postherpetic neuralgia, and other causes (Richardson et al., 2010). The incidence of PN attributed to MM treatment varies by class of drug, by agent and dosing schedule (Table 9). Baseline evaluation of each patient is critical to identify contributing factors that may be reversed, and to implement well-established guidelines for dose modification or discontinuation of agents that are associated with more severe PN. Given the improved trends in survival and the expanded options for treatment, irreversible and debilitating PN is an unacceptable outcome. Refinement of dosing and scheduling of novel agents for MM has improved the incidence and severity of PN, however, health-care providers must be able to identify patients at increased risk, establish a standard for evaluating the onset or progression of PN; and be familiar with the established guidelines for dose modification or in some cases selection of therapies for patients with MM and existing PN.

Thromboembolic events, including deep-vein thrombosis and pulmonary emboli, present an additional challenge in the treatment of MM. Multiple myeloma itself is a risk factor for thrombosis (Palumbo et al., 2008). Evaluation of each patient for additional risk factors for thrombosis is essential prior to thalidomide, lenalidomide, and pomalidomide therapy. All patients treated with these agents require thromboprophylaxis based on established guidelines.

The incidence of herpes zoster virus (HSV) reported with bortezomib is variable but was notably decreased when antiviral prophylaxis was mandated in the VISTA trial (Mateos et al., 2006). It is important to remember that antiviral medications require dose modification for renal impairment and should be dosed based upon creatinine clearance. Although there was no incidence of HSV reported in the PX-171-003-A1 trial with carfilzomib, antiviral prophylaxis was only required for patients with a history of herpes zoster or simplex.

Alteration in hemodynamics ranges from hypotension to hypertension, with both being reported in approximately 14% of patients receiving bortezomib, and hypertension alone reported for approximately 14% with carfilzomib (Millennium, 2012; Onyx Pharmaceuticals, 2012). As the average age at diagnosis for MM is 69 years, cardiac comorbidities are common, and blood pressure management should incorporate a collaborative approach. Safety to avoid falls from orthostatic hypotension is a priority for patient education followed by modification of antihypertensive therapy.

## Conclusion

The integration of novel agents into the treatment of MM has shifted the focus from an incurable disease to a disease that is chronic with a realistic hope of long-term survival and quality of life. Proteasome inhibitors and immunomodulatory agents are the backbone of novel therapies for the treatment of MM. Recent trials and next generation agents are particularly important for patients with relapsed or refractory disease, a fate faced by the majority of myeloma patients over the course of their disease. The improvement in overall survival reported with proteasome inhibitors and immunomodulatory agents illustrates the efficacy of these agents, the importance of early identification and management of treatment-related toxicities, and the significant contribution of clinical trials participation. Despite these exciting developments, MM patients continue to succumb to their disease and experience adverse events related to their disease and treatment. Continued patient enrollment in clinical trials including genomic analysis will be necessary to fully characterize and exploit targets within the malignant clone and the tumor microenvironment necessary to identify new agents to complement the existing novel therapies. The hope of someday finding a cure for this disease will require ongoing research.

Evolving strategies that include the combination of multiple agents to attack alternative pathways have improved clinical outcomes. These combinations offer significant promise to patients diagnosed with MM but also present a number of challenges. As treatment strategies are combined adverse events profiles will change. The numerous trials conducted throughout the world with variable trial design, patient populations, and clinical trial endpoints have produced a plethora of data that are difficult to consolidate into tangible clinical recommendations. Although algorithms for risk-adapted treatment of MM have been developed, a lack of consensus exists as to the optimal combination and sequencing of therapies, and the role and timing for autologous stem cell transplantation. We are fortunate to have so many good options for treatment of MM; however, this presents a challenge to patients and caregivers who may receive conflicting recommendations from various providers. Global working groups, such as the International Myeloma Foundation Working Group, have embarked on efforts to identify priorities for continued research initiatives and collaborative efforts to utilize existing data sources to maximize the benefit to patients and to future research.

The advanced practitioner in oncology plays an integral role in the early identification and clinical management of common adverse events; reporting of less common side effects that may not have been reported in clinical trials, and education of patients and caregivers about their disease, their individual treatment plan, and how they can take an active role in self-management of and reporting of adverse events. The application of advances in science together with effective clinical management and formation of a partnership with MM patients and their caregivers will provide the best opportunity for continued treatment and favorable clinical outcomes.
